# Supplementary material for: Role and mechanism of NCAPD3 in promoting malignant behaviors in gastric cancer
Source: Front Pharmacol. 2024 Apr 22;15:1341039. doi: 10.3389/fphar.2024.1341039 (PMC11070777; doi:10.3389/fphar.2024.1341039)
Supplement: Supplementary file 11 [file DataSheet2.ZIP › GSEA/Transcipt factor/my_analysis.Gsea.1599461814734/FOXO4_02.html]

Details for gene set FOXO4\_02[GSEA]

|  || Dataset | filtered\_dataset.sample\_info.cls#WT\_versus\_NCAPD3\_MUT |
| Phenotype | sample\_info.cls#WT\_versus\_NCAPD3\_MUT |
| Upregulated in class | NCAPD3\_MUT |
| GeneSet | FOXO4\_02 |
| Enrichment Score (ES) | -0.39098194 |
| Normalized Enrichment Score (NES) | -2.1214542 |
| Nominal p-value | 0.0 |
| FDR q-value | 0.04097961 |
| FWER p-Value | 0.301 |
Table: GSEA Results Summary

  

Fig 1: Enrichment plot: FOXO4\_02      
 Profile of the Running ES Score & Positions of GeneSet Members on the Rank Ordered List

  

| SYMBOL | TITLE | RANK IN GENE LIST | RANK METRIC SCORE | RUNNING ES | CORE ENRICHMENT || 1 | 23338 | JADE2 | 71 | 0.855 | -0.0014 | No |
| 2 | 221504 | ZBTB9 | 237 | 0.647 | -0.0830 | No |
| 3 | 152485 | ZNF827 | 268 | 0.618 | -0.0685 | No |
| 4 | 4297 | KMT2A | 337 | 0.573 | -0.0842 | No |
| 5 | 4784 | NFIX | 567 | 0.439 | -0.2245 | No |
| 6 | 84335 | AKT1S1 | 572 | 0.437 | -0.2018 | No |
| 7 | 26100 | WIPI2 | 601 | 0.423 | -0.1973 | No |
| 8 | 11083 | DIDO1 | 631 | 0.406 | -0.1945 | No |
| 9 | 149076 | ZNF362 | 680 | 0.388 | -0.2066 | No |
| 10 | 7357 | UGCG | 852 | -0.254 | -0.3156 | No |
| 11 | 10123 | ARL4C | 957 | -0.377 | -0.3689 | Yes |
| 12 | 7067 | THRA | 976 | -0.392 | -0.3590 | Yes |
| 13 | 9572 | NR1D1 | 977 | -0.392 | -0.3360 | Yes |
| 14 | 51667 | NUB1 | 978 | -0.392 | -0.3130 | Yes |
| 15 | 10938 | EHD1 | 1019 | -0.419 | -0.3174 | Yes |
| 16 | 7871 | SLMAP | 1038 | -0.435 | -0.3050 | Yes |
| 17 | 23764 | MAFF | 1039 | -0.436 | -0.2794 | Yes |
| 18 | 2012 | EMP1 | 1119 | -0.494 | -0.3077 | Yes |
| 19 | 7074 | TIAM1 | 1143 | -0.508 | -0.2946 | Yes |
| 20 | 639 | PRDM1 | 1159 | -0.523 | -0.2748 | Yes |
| 21 | 27250 | PDCD4 | 1170 | -0.537 | -0.2506 | Yes |
| 22 | 2119 | ETV5 | 1176 | -0.545 | -0.2222 | Yes |
| 23 | 6310 | ATXN1 | 1177 | -0.547 | -0.1902 | Yes |
| 24 | 153222 | CREBRF | 1187 | -0.559 | -0.1639 | Yes |
| 25 | 1358 | CPA2 | 1263 | -0.648 | -0.1803 | Yes |
| 26 | 54800 | KLHL24 | 1287 | -0.677 | -0.1572 | Yes |
| 27 | 861 | RUNX1 | 1293 | -0.687 | -0.1206 | Yes |
| 28 | 3084 | NRG1 | 1320 | -0.739 | -0.0962 | Yes |
| 29 | 113791 | PIK3IP1 | 1345 | -0.787 | -0.0674 | Yes |
| 30 | 4734 | NEDD4 | 1369 | -0.856 | -0.0339 | Yes |
| 31 | 26959 | HBP1 | 1395 | -1.073 | 0.0109 | Yes |
Table: GSEA details [plain text format]

  

Fig 2: FOXO4\_02      
 Blue-Pink O' Gram in the Space of the Analyzed GeneSet

  

Fig 3: FOXO4\_02: Random ES distribution      
 Gene set null distribution of ES for **FOXO4\_02**
